# White Matter Microstructural Abnormalities in Neonatal Onset Genetic Epilepsy

**DOI:** 10.1002/acn3.70440

**Published:** 2026-05-22

**Authors:** Amanda G. Sandoval Karamian, Tianjia Zhu, Hao Huang, Shavonne L. Massey, Mark P. Fitzgerald, Darshana Parikh, Arastoo Vossough, Ingo Helbig, Nicholas S. Abend

**Affiliations:** ^1^ Division of Neurology, Department of Pediatrics University of Utah School of Medicine Salt Lake City Utah USA; ^2^ Department of Radiology Children's Hospital of Philadelphia Philadelphia Pennsylvania USA; ^3^ Department of Bioengineering, School of Engineering and Applied Science University of Pennsylvania Philadelphia Pennsylvania USA; ^4^ Department of Radiology Perelman School of Medicine, University of Pennsylvania Philadelphia Pennsylvania USA; ^5^ Division of Neurology, Department of Pediatrics Children's Hospital of Philadelphia Philadelphia Pennsylvania USA; ^6^ Departments of Neurology and Pediatrics University of Pennsylvania Perelman School of Medicine Philadelphia Pennsylvania USA; ^7^ Epilepsy Neurogenetics Initiative (ENGIN) Children's Hospital of Philadelphia Philadelphia Pennsylvania USA

**Keywords:** genetic epilepsy, neonatal seizures, white matter

## Abstract

**Objective:**

Recent evidence indicates that epilepsy is associated with abnormal white matter. If seizures alter white matter, then the impact upon network function, epileptogenesis, and cognition could be pronounced in neonates undergoing rapid developmental myelination. Neonates with epilepsy due to nonstructural genetic causes provide a unique opportunity to determine whether neonates experiencing seizures have abnormal white matter structure.

**Methods:**

This was a retrospective case–control study of term neonates treated in a level IV NICU between 2013 and 2020. Cases had confirmed or suspected genetic epilepsy, normal MRI, and no conditions known to independently impact white matter. Healthy controls had normal MRI and no relevant clinical diagnoses. White matter was assessed via fractional anisotropy (FA) and mean diffusivity (MD) from Diffusion Tensor Imaging (DTI) using Tract Based Spatial Statistics (TBSS).

**Results:**

Fifty‐eight neonates (19 cases, 39 controls) were included. There was significantly increased FA and decreased MD in the superior corona radiata of neonates with genetic epilepsy compared to healthy controls, controlling for sex and postmenstrual age at time of MRI. Additional association tracts (anterior and posterior corona radiata, tapetum, external capsule, superior and inferior fronto‐occipital fasciculus) approached significance (*p* < 0.2). There was no significant correlation between mean FA or MD and EEG seizure burden or developmental outcome.

**Interpretation:**

White matter microstructure is abnormal, with higher FA and lower MD, in major association tracts of neonates with genetic epilepsy compared to healthy controls. These results indicate that white matter is impacted early in neonatal epilepsies, emphasizing the global impact of early‐onset seizures.

## 1 Introduction

Structural changes in neural networks may contribute to epileptogenesis and cognitive dysfunction in children with epilepsy, although the pathophysiology remains unclear [1, 2, 3]. A largely unexplored possibility is that seizures alter white matter. Animal and human studies indicate that white matter is dynamic during and after developmental myelination. Changes are driven by spontaneous and stimulated neuronal activity [4, 5], a process required for multiple forms of learning [4, 5, 6, 7, 8, 9, 10, 11]. Animal models indicate that the neuronal activity of seizures also alters myelin [5] which may impact subsequent seizure frequency and cognition [6, 12, 13, 14].

Diffusion tensor imaging (DTI) enables quantitative assessment of white matter in humans. DTI assesses mean diffusivity (MD) which is independent of direction and fractional anisotropy (FA) which indicates the orientation of diffusion [15]. MD and FA are inversely and directly correlated with white matter structural integrity, respectively [16]. Changes in MD and FA have been demonstrated in temporal lobe epilepsy, frontal lobe epilepsy, and generalized epilepsy in adults [17, 18]. Further, changes in MD and FA have been demonstrated in children with temporal lobe epilepsy [19, 20], benign epilepsy with centrotemporal spikes [21], and idiopathic epilepsy [22, 23]. Additionally, accelerated myelination has been described in infants with hemimegalencephaly in the abnormal hemisphere and in some cases extending into the contralateral hemisphere via the corpus callosum [24]. These findings indicate that abnormal white matter may contribute to cognitive impairment and developmental delay comorbid with many forms of pediatric epilepsy [25]. Altered white matter could also impact seizure frequency [6].

If seizures alter white matter, then the impact of this interaction upon epileptogenesis and cognitive function could be particularly pronounced in neonates and infants experiencing seizures during the time period when they are also undergoing rapid developmental myelination [26]. In neonates, white matter is present in an immature state in the major tracts of the brain (including the corpus callosum and internal capsules) [26, 27]. Although white matter development is incomplete, these tracts show measurable MD and FA [16, 26]. To our knowledge, studies have not systematically assessed for an association between neonatal seizures and altered white matter using DTI.

Genetic epilepsy is a major cause of seizures during the newborn period and provides an opportunity to address this question [28]. Other causes of neonatal seizures, such as hypoxic–ischemic encephalopathy, stroke, infection, intraventricular hemorrhage, and major structural malformations likely lead to altered white matter through mechanisms independent of seizures. Multiple etiologic genetic mutations have been identified in neonates with epilepsy [28, 29], and many neonatal onset genetic epilepsies cause seizures but are not known to impact white matter structure on conventional neuroimaging. However, when looking more closely, some differences can be appreciated. For example, previous preliminary investigations of white matter microstructural abnormalities in neonates with epilepsy due to diverse genetic causes suggested convergent corpus callosum microstructural abnormalities using MD from apparent diffusion coefficient (ADC) maps [30].

The goal of this study was to determine whether white matter was abnormal in neonates with genetic epilepsy using DTI, and also assess whether seizure burden impacted white matter abnormalities. We hypothesized that neonatal seizures occurring during rapid developmental myelination are associated with measurable disruption of white matter microstructure. Specifically, that neonates with genetic epilepsy would have higher FA and lower MD in major white matter tracts compared with age‐matched controls, and that increasing EEG seizure burden would correlate with greater differences in FA and MD.

## 2 Materials and Methods

### 2.1 Standard Protocol Approvals, Registrations, and Patient Consents

This study was approved by the Institutional Review Board of the Children's Hospital of Philadelphia (CHOP), Federalwide Assurance #00000459. A waiver of participant consent was determined to be appropriate since all data used in this study were obtained either as part of routine clinical care or from prior research studies.

### 2.2 Study Design

This was a retrospective case–control study of neonates with genetic epilepsy treated in the Neonatal Intensive Care Unit at CHOP between 2013 and 2020.

### 2.3 Cohort Development

Case and control cohorts were established. Inclusion criteria were term neonates with gestational age ≥ 36 weeks and post menstrual age ≤ 44 weeks who underwent DTI in the neonatal period.

#### 2.3.1 Cases With Genetic Epilepsy

Neonates were identified from the CHOP Neonatal Seizure Database and a database of neonates with genetic testing performed for epilepsy through CHOP's Epilepsy Neurogenetics Initiative (ENGIN). Cases were neonates with confirmed or suspected genetic epilepsy, normal brain MRI, and no other conditions expected to independently affect white matter (neonates with congenital heart disease, exposure to medications known to alter white matter (i.e., antidepressants, stimulants), meningitis, stroke, and hypoxic–ischemic encephalopathy were excluded). Due to the relative rarity of these mutations and the small numbers of neonates with each mutation, cases were combined into a single group for analysis.

#### 2.3.2 Controls

There were two control groups. *Clinical Controls* were healthy neonates with a normal MRI which had been obtained for clinical purposes while in the CHOP NICU. Exclusion criteria included abnormal MRI or conditions known to cause alterations in white matter structure, including congenital heart disease, exposure to medications known to alter white matter (i.e., antidepressants, stimulants), meningitis, stroke, and hypoxic–ischemic encephalopathy. *Research Controls* were obtained as part of a prior research study to establish an age‐specific neonatal DTI atlas [31]. The clinical and research control subjects were combined into a single control group for analysis.

Cases and controls were age‐matched for gestational age at birth.

### 2.4 Imaging Analysis

#### 2.4.1 Cases and Clinical Controls

Neonates underwent 3 Tesla diffusion MRI (dMRI). DMRI sequences were obtained as part of the clinically indicated MRI acquired from 3 Tesla Siemens MRI (Verio and Skyra) scanners (Siemens, Erlangen, Germany). The imaging parameters were: TE = 65–78 ms, TR = 5780–6850 ms, in‐plane imaging resolution = 2 × 2 mm^2^, slice thickness = 2.5 mm, 30 independent diffusion encoding directions, *b* = 1000 s/mm^2^. Only images with less than 5 volumes affected by motion were accepted. DTI was processed using DTIStudio (mristudio.org). All DWIs were registered to the b0 image using a 12‐parameter affine transformation for correction of motion and distortion caused by eddy currents. Diffusion tensor fitting was conducted with corrected DWIs. DTI‐derived FA and MD maps were obtained after tensor fitting. The FA and MD images were upsampled to match the resolution of the research control dMRI data (0.6 mm isotropic). Imaging analysis was performed by ASK and TZ, who were masked to the clinical case/control status.

#### 2.4.2 Research Controls

DTI for research controls was obtained as part of a prior research study to establish an age‐specific neonatal DTI atlas [31]. Image acquisition and parameters for the research controls were previously described [31]. Briefly, Diffusion MRI (dMRI) was acquired from a 3 Tesla Philips Achieva MR system with imaging parameters: TE = 78 ms, TR = 6850 ms, in‐plane field of view = 168 × 168 mm^2^, in‐plane imaging resolution = 1.5 × 1.5 mm^2^, slice thickness = 1.6 mm, slice number = 60, 30 independent diffusion encoding directions, *b* = 1000 s/mm^2^, and repetition = 2. All DTI‐derived maps were then resliced to 0.6 mm isotropic resolution with a 180 × 220 × 180 matrix to the same resolution as the age‐specific neonate atlas.

#### 2.4.3 Atlas‐Based Tract Level Quantification of DTI‐Derived Metrics on White Matter Skeleton

All FA and MD values were measured at the core white matter in the template space [32, 33, 34, 35, 36, 37] of the age‐specific 39 week neonate atlas [31]. All white matter tracts within the neonate atlas [31] were analyzed, 53 tracts in total. Data harmonization was conducted with ComBat [38] based on the FA and MD measured at core white matter described above.

### 2.5 Clinical Data and Outcomes

Demographic and clinical data were collected from chart review of the electronic medical record, including gestational age, sex, birth weight, age at time of hospital admission, Apgar scores, neurological examinations, number of anti‐seizure medications (ASM) administered, genetic testing results, and age and postmenstrual age at time of MRI. Data were compiled and securely stored in a Research Electronic Data Capture (REDCap) database hosted at CHOP [39, 40].

A neonatal presentation severity category was created for descriptive characterization of the cohort based on neurologic examination severity, EEG background category, and number of ASMs administered. This investigator‐defined summary variable was not intended as a validated clinical severity scale and was not used as a primary predictor in the diffusion analyzes. Neonatal presentation severity for cases was grouped into three categories based on neurological examination, number of ASM administered, and EEG background category. The neurologic examination was adapted from the modified Sarnat examination and categorized as normal or mildly abnormal, moderately abnormal (moderate in 2–4 categories or moderate in 2 and severe in 1 categories), or severely abnormal (moderate in 5–6 categories or moderate in 3 and severe in 1 category or severe in ≥ 2 categories). The number of ASMs was determined by the number of unique medications given for seizure control, including both loading bolus doses and maintenance doses (i.e., if phenobarbital was given as both a loading dose and maintenance medication, this would count as 1 ASM, but if phenobarbital and fosphenytoin were given as loading doses with only phenobarbital as a maintenance medication, this would count as 2 ASMs). EEG background was categorized as normal or mildly abnormal, moderately abnormal, or severely abnormal as summarized below.

*Mild Presentations* included a normal or mildly abnormal neurological exam, 1 ASM, and a normal or mildly abnormal EEG background. *Moderate Presentations* included a moderately abnormal neurological examination, 2–3 ASMs, and a moderately abnormal EEG background. *Severe Presentations* included a severely abnormal neurological exam, > 3 ASMs, and a severely abnormal EEG background.

Development was categorized as typical or delayed based on information available in the electronic medical record from outpatient visits with Neurology, Developmental and Behavioral Pediatrics, and Rehabilitation Medicine, including Physical, Occupational, and Speech Therapy. Subjects were categorized as delayed if developmental delay was noted in any category (gross motor, fine motor, speech/language, social/cognitive) at any follow‐up visit.

### 2.6 EEG Analysis

Complete EEG tracings were available for all cases. EEG tracings were not performed/available for clinical controls or research controls. Neonatal EEG background scoring was adapted from Clancy et al. [41] Background scoring was performed for a one‐hour period at 0, 6, 12, 18, 24, 36, 48, 72, and 96 h after the start of the recording for each subject by a pediatric electroencephalographer (A.S.K.) blind to outcome data.

EEG background scoring was incorporated into the neonatal presentation severity scoring as above. Scoring included an overall background category: (1) normal; (2) mildly abnormal (mildly excessive discontinuity with inter‐burst intervals < 10 s, mildly excessive interhemispheric asynchrony, poor concordance between clinical and electrographic sleep states, mild poverty of anticipated background rhythms, mild focal abnormalities such as excessive sharp waves or focal voltage attenuation); (3) moderately abnormal (moderately excessive discontinuity with inter‐burst intervals 10–30 s, moderately excessive interhemispheric asynchrony, poverty of anticipated background rhythms, definite focal abnormalities such as persistent focal delta activity or focal depression of expected background patterns, persistent low voltage < 25uV for all states); (4) and severely abnormal (markedly excessive discontinuity for age with inter‐burst intervals > 30 s, burst suppression pattern, severe interhemispheric asynchrony, low voltage suppression < 10uV).

Seizures were defined according to the current American Clinical Neurophysiology Society guidelines on neonatal EEG [42]. Seizure burden prior to the MRI was assessed as: (1) the number of seizures, (2) total number of seconds of seizures, and (3) total duration of seizures categorized as low (< 5 min), medium (5–12 min), and high (≥ 12 min) seizure burden. These measures were selected to capture seizure frequency, cumulative seizure exposure, and a clinically interpretable burden grouping, respectively.

### 2.7 Statistical Analysis

Mean MD and FA measurements were first compared between the case (genetic epilepsy) and control (combined clinical and research controls) groups using Mann–Whitney *U* tests as bivariate analyzes, with correction for multiple comparisons. To account for potential confounding, adjusted analyzes were then performed using linear regression models with MD or FA as the dependent variable and case–control status as the independent variable, adjusting for sex and postmenstrual age at MRI. Among cases, associations between seizure burden and MD/FA were assessed using three measures of seizure burden prior to MRI: (1) total number of seizures, (2) cumulative seizure duration in seconds, and (3) seizure burden category (low, medium, high as described above). Unadjusted associations were assessed using Spearman's rank correlation for continuous seizure‐burden measures and Kruskal‐Wallis testing for categorical seizure‐burden groups. Adjusted analyzes were performed using linear regression models controlling for postmenstrual age at MRI. *T*‐tests and point biserial correlation were used to determine whether mean MD and FA were correlated with developmental outcome (normal development versus developmental delay). A statistical significance was set at *p* < 0.05.

Exploratory subgroup analyzes were performed within the genetic epilepsy cohort to compare diffusion metrics between (1) KCNQ2/3‐related epilepsy versus other genetic or presumed genetic etiologies, and (2) cases without a pathogenic etiology identified on genetic testing versus cases with an identified genetic etiology. An additional control subgroup analysis compared clinical controls versus research controls to assess for within‐control‐group differences. Subgroup analyzes applied both unadjusted analyzes and adjusted linear regression models controlling for postmenstrual age at MRI and sex. Given the small sample sizes, these analyzes were considered exploratory and descriptive statistics are emphasized.

## 3 Results

### 3.1 Subject Characteristics

A total of 19 cases and 39 controls (7 clinical controls and 32 research controls) were analyzed. Subject characteristics are summarized in Table 1. Reason for NICU admission for the 7 clinical controls included concern for vocal fold hypomobility (2), periodic breathing (1), reflux/BRUE (1), benign paroxysmal downgaze (2), and poor feeding which resolved (1). Controls were significantly older than cases at the time of MRI. However, there was no difference in the post menstrual age at MRI, which is the relevant age measure for white matter maturity at the time of MRI. There were no other demographic differences between cases and controls.

**TABLE 1:** Subject characteristics.

Variable	Controls (*n* = 39)	Cases (*n* = 19)	*p*
Gestational age at birth, weeks, median (IQR)	40.0 (38.9, 40.6)	39.4 (38.5, 40.5)	0.61
Male sex, *n* (%)	27 (69.0)	11 (57.9)	0.48
Birth weight, kg, median (IQR)	3.6 (3.4, 3.8)	3.4 (3.0, 3.8)	0.44
Age at MRI, days, median (IQR)	17.5 (8.5, 26.0)	7 (5, 10.5)	**0.02**
Post menstrual age at MRI, weeks, mean (SD)	42.0 (1.7)	40.9 (1.8)	0.16
	**Controls (*n* = 7)**	**Cases (*n* = 19)**	
Apgar score at 1 min, median (IQR)^a^	8 (8, 8)	8 (8, 9)	0.16
Apgar score at 5 min, median (IQR)^a^	9 (9, 9)	8 (8, 9)	0.16
Age at admit to NICU, days, median (IQR)^a^	14 (2, 25)	5 (4, 9)	0.17

*Note:* Significant *p*‐values < 0.05 are in bold.

^a^ Data available for only the clinical control group.

Among cases with genetic epilepsy, 12 (63%) had channelopathies, including 10 subjects with *KCNQ2*‐related epilepsy. In 6 of the cases (32%), there was no etiology identified on genetic testing and no other cause for seizures identified after comprehensive diagnostic evaluation; therefore, these neonates were considered to be genetic in etiology but not detected by current testing methods. Clinical characteristics of the genetic epilepsy cohort are summarized in Table 2. These included neurologic examination severity, EEG background category, number of anti‐seizure medications administered, seizure etiology, and developmental outcome. None of the cases had a severe neonatal presentation. Nine cases (47%) had developmental delay in any domain.

**TABLE 2:** Seizure etiologies, severity of neonatal presentation, and developmental outcome in cases.

Gene	*n*	Neonatal presentation^a^			Developmental outcome^b^	
		Mild	Moderate	Severe	Typical	Delayed
*KCNQ2*	10	7	3		6	4
*SCN2A*	1		1			1
*KCNQ3*	1	1			1	
*TCF4*	1		1			1
No etiology identified on genetic testing	6	4	2		3	3

^a^ Neonatal presentation categorized as mild, moderate, or severe based on neurological examination, number of antiseizure medications, and EEG background (see Methods section).

^b^ Median duration of follow‐up 21 months, IQR (10, 34).

### 3.2 Imaging Analysis

**FIGURE 1:**
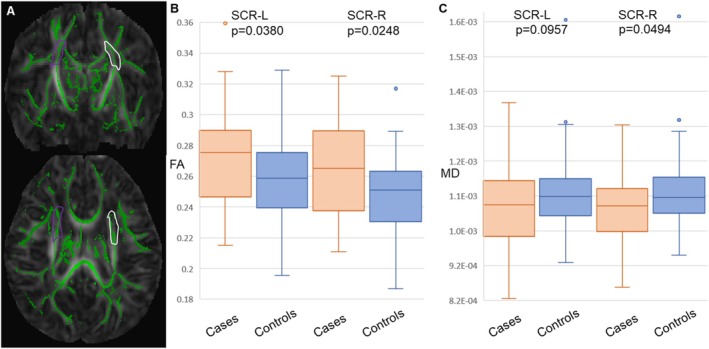
White matter tracts analyzed with significant differences in mean fractional anisotropy (FA) and mean diffusivity (MD) between cases and controls. (A) Representative outline of the Superior Corona Radiata (SCR) at one level on a coronal and axial FA map. (B) Box and whisker plots showing significantly higher FA in cases vs. controls in the right and left SCR. (C) Box and whisker plots showing significantly lower MD in cases vs. controls in the right SCR, with a non‐significant decrease in MD in the left SCR.

FA and MD were analyzed for 53 white matter tracts from DTI using tract based spatial statistics. Mean FA was significantly higher in cases versus controls in the left superior corona radiata (*p* = 0.017) and right superior corona radiata (*p* = 0.017) (Figure 1). For exploratory analysis, regions with *p*‐values < 0.2 included the right posterior corona radiata (*p* = 0.160), left tapetum (*p* = 0.065), right tapetum (*p* = 0.072), left external capsule (*p* = 0.077), right external capsule (*p* = 0.115), and left middle cerebellar peduncle (*p* = 0.148). Selected relevant findings are presented in Table 3, with mean FA values for each region for cases and controls summarized in Table S1.

**TABLE 3:** Mean fractional anisotropy (FA) values for cases and controls for selected white matter tracts analyzed. Complete data for all 53 tracts analyzed are included in Table S1.

Structure	Laterality	Mean (SD) FA cases	Mean (SD) FA controls	*p*
Corpus callosum	Left	0.411 (0.044)	0.407 (0.041)	0.5891
Corpus callosum	Right	0.406 (0.044)	0.405 (0.036)	0.6096
Posterior limb of internal capsule	Left	0.497 (0.030)	0.499 (0.029)	0.9841
Posterior limb of internal capsule	Right	0.497 (0.029)	0.503 (0.029)	0.9543
Superior corona radiata	Left	0.275 (0.035)	0.258 (0.030)	**0.0166**
Superior corona radiata	Right	0.266 (0.029)	0.249 (0.026)	**0.0169**
Posterior corona radiata	Left	0.288 (0.030)	0.281 (0.030)	0.2138
Posterior corona radiata	Right	0.294 (0.026)	0.284 (0.031)	0.1594
Tapetum	Left	0.287 (0.038)	0.270 (0.043)	0.0647
Tapetum	Right	0.288 (0.030)	0.271 (0.039)	0.0724
External capsule	Left	0.280 (0.022)	0.271 (0.021)	0.0772
External capsule	Right	0.274 (0.022)	0.266 (0.020)	0.1145
Superior cerebellar peduncle	Left	0.315 (0.023)	0.321 (0.028)	0.6960
Superior cerebellar peduncle	Right	0.321 (0.024)	0.325 (0.031)	0.9976
Middle cerebellar peduncle	Left	0.320 (0.037)	0.318 (0.038)	0.1478
Middle cerebellar peduncle	Right	0.319 (0.043)	0.322 (0.035)	0.9872
Inferior cerebellar peduncle	Left	0.288 (0.038)	0.291 (0.036)	0.8442
Inferior cerebellar peduncle	Right	0.290 (0.043)	0.297 (0.039)	0.9039
Pontine crossing tract	Left	0.259 (0.028)	0.263 (0.031)	0.4748
Pontine crossing tract	Right	0.250 (0.035)	0.245 (0.029)	0.8381

*Note:* Significant *p*‐values < 0.05 are in bold.

MD was significantly lower in cases versus controls in the right superior corona radiata (*p* = 0.049) (Figure 1). For exploratory analysis, regions with *p*‐values < 0.2 included the left superior corona radiata (*p* = 0.096), left anterior corona radiata (*p* = 0.124), right anterior corona radiata (*p* = 0.106), right posterior corona radiata (*p* = 0.147), left tapetum (*p* = 0.115), right tapetum (*p* = 0.100), left external capsule (*p* = 0.153), right external capsule (*p* = 0.156), right superior fronto‐occipital fasciculus (*p* = 0.184), right inferior fronto‐occipital fasciculus (*p* = 0.191), left pontine crossing tract (*p* = 0.140), and right pontine crossing tract (*p* = 0.102). Selected relevant findings are presented in Table 4, with mean MD values for each region for cases and controls summarized in Table S2.

**TABLE 4:** Mean diffusivity (MD) values for cases and controls for selected white matter tracts analyzed. Complete data for all 53 tracts analyzed are included in Table S2.

Structure	Laterality	Mean (SD) MD cases	Mean (SD) MD controls	*p*
Corpus callosum	Left	0.00100 (0.00013)	0.00101 (0.00012)	0.3443
Corpus callosum	Right	0.00101 (0.00013)	0.00101 (0.00012)	0.3095
Posterior limb of internal capsule	Left	0.00071 (0.00007)	0.00071 (0.00008)	0.9914
Posterior limb of internal capsule	Right	0.00071 (0.00006)	0.00071 (0.00008)	0.9670
Superior corona radiata	Left	0.00109 (0.00014)	0.00113 (0.00012)	0.0957
Superior corona radiata	Right	0.00109 (0.00012)	0.00114 (0.00012)	**0.0494**
Posterior corona radiata	Left	0.00108 (0.00012)	0.00109 (0.00011)	0.3041
Posterior corona radiata	Right	0.00108 (0.00011)	0.00110 (0.00012)	0.1472
Tapetum	Left	0.00126 (0.00015)	0.00130 (0.00016)	0.1152
Tapetum	Right	0.00123 (0.00012)	0.00127 (0.00015)	0.0998
External capsule	Left	0.00100 (0.00008)	0.00103 (0.00010)	0.1525
External capsule	Right	0.00101 (0.00007)	0.00104 (0.00010)	0.1557
Superior cerebellar peduncle	Left	0.00081 (0.00007)	0.00081 (0.00009)	0.7784
Superior cerebellar peduncle	Right	0.00080 (0.00008)	0.00079 (0.00010)	0.8991
Middle cerebellar peduncle	Left	0.00096 (0.00014)	0.00095 (0.00014)	0.8528
Middle cerebellar peduncle	Right	0.00097 (0.00013)	0.00094 (0.00013)	0.7424
Inferior cerebellar peduncle	Left	0.00093 (0.00016)	0.00090 (0.00015)	0.6807
Inferior cerebellar peduncle	Right	0.00089 (0.00014)	0.00088 (0.00013)	0.7201
Pontine crossing tract	Left	0.00090 (0.00017)	0.00095 (0.00017)	0.1403
Pontine crossing tract	Right	0.00094 (0.00021)	0.00100 (0.00019)	0.1023

*Note:* Significant *p*‐values < 0.05 are in bold.

Two subgroup analyzes were performed. The first analysis assessed for differences between *KCNQ2/3* and other genetic etiologies, and there were no significant differences in mean FA or MD. The second analysis assessed for differences between cases with no etiology identified on genetic testing and cases with an identified genetic etiology, and there were no significant differences in mean FA or MD. Additionally, an analysis was performed to compare FA and MD between clinical controls and research controls to ensure there were no within‐group differences in the control group as a whole, with no significant differences in mean FA or MD.

### 3.3 EEG Analysis

Among neonates with genetic epilepsy, seizure burden prior to MRI was summarized in three ways: total number of seizures (median 3.5, IQR 3.0–8.3), total seizure duration in seconds (median 268.5, IQR 173.0–482.5), and seizure burden category (low < 5 min, *n* = 11; medium 5–12 min, *n* = 5; high ≥ 12 min, *n* = 3).

Controlling for postmenstrual age at MRI and sex, there were no associations between seizure burden and FA or MD (β range −0.00126 to 0.00060, CI range −0.00280 to 0.00210, all *p* > 0.05). When seizure burden was analyzed as total number of seizures, there were no significant associations with FA or MD (β range −0.00126 to 0.00060, 95% CI range −0.00280 to 0.00210, all *p* > 0.05). When seizure burden was assessed as cumulative seizure duration, there were no significant associations with FA or MD (β range −4.77 × 10^−7^ to 2.86 × 10^−6^, 95% CI range −3.10 × 10^−6^ to 3.50 × 10^−6^, all *p* > 0.05). When seizure burden was assessed as a categorical variable, there were no significant associations with FA or MD (β range −0.0155 to 0.0115, 95% CI range −0.040 to 0.030, all *p* > 0.05).

Because the sample size was limited, we performed a post hoc sample size analysis, which suggested that approximately 88 subjects would be needed to detect an association of the observed magnitude between mean FA and seizure burden. This finding is presented to contextualize the negative result rather than to imply that limited power definitively explains the absence of association.

### 3.4 Developmental Outcomes

Developmental outcome data are summarized in Table 2. Nine of 19 cases (47%) demonstrated developmental delay in at least one domain at follow‐up. In secondary analyzes comparing cases with typical versus delayed development, mean FA in the right hippocampal portion of the cingulum differed significantly between groups (*p* = 0.001). No other regions demonstrated significant differences in FA or MD. Given the small sample size and multiple regional comparisons, this finding should be interpreted cautiously.

### 3.5 Subgroup Analysis

Exploratory subgroup analyzes comparing neonates with KCNQ2/KCNQ3‐related epilepsy to those with other genetic or presumed genetic etiologies demonstrated no significant differences in FA or MD. This finding was consistent in both unadjusted analyzes and adjusted linear regression models controlling for postmenstrual age at MRI and sex. Subgroup analysis results are provided in Table S3.

Additional subgroup analyzes comparing clinical controls versus research controls demonstrated no significant differences in FA or MD. This finding was consistent in both unadjusted analyzes and adjusted linear regression models controlling for postmenstrual age at MRI and sex.

## 4 Discussion

White matter microstructure is abnormal, with higher FA and lower MD in major tracts of neonates with epilepsy due to diverse genetic causes compared to healthy controls when controlling for sex and postmenstrual age at time of MRI. FA is directly correlated with white matter structural integrity and indicates orientation of diffusion; higher FA in cases suggests aberrantly increased white matter structural integrity in cases [15, 16]. MD is inversely correlated with white matter structural integrity and is independent of diffusion direction; lower MD in cases also supports abnormally increased white matter structural integrity in neonates with genetic epilepsy [15, 16]. These findings are concordant with prior findings of decreased MD in a major commissural tract, the corpus callosum, in neonatal genetic epilepsy [30]. Based on the principle of activity‐dependent myelination, the excessive neuronal activity of seizures may impact white matter structure and lead to aberrant network formation; however, significant associations between seizure burden and diffusion measures were not identified in this cohort. Given the modest sample size, this negative finding may reflect either a true absence of association or limited power to detect a relationship of small to moderate magnitude. Accordingly, these data do not definitively support strong mechanistic conclusions regarding activity‐dependent myelination or other seizure‐related effects on white matter development. These possibilities warrant investigation in larger cohorts with prospectively standardized EEG and imaging measures.

There was a single region bilaterally, the superior corona radiata, that was significantly different between cases and controls for both FA and MD. The superior corona radiata is a major projection tract connecting the cerebral cortex to the brainstem. The corona radiata has been implicated in several neurocognitive abnormalities, with lower FA reported in the anterior corona radiata of patients with bipolar disorder with depressive episodes, and lower FA in the corona radiata of patients with schizophrenia [43, 44, 45]. White matter abnormalities have also been reported in the corona radiata of children with ADHD [46, 47]. Ischemic strokes in the corona radiata have demonstrated functional network changes on fMRI, and ischemic injury of the corona radiata has been associated with poor gait recovery in adults after stroke [48, 49]. These data suggest the involvement of the corona radiata in broader network function contributing to neurocognitive and motor outcomes.

Exploratory analyzes found higher mean FA (*p* < 0.2) in the posterior corona radiata, tapetum, external capsule, and middle cerebellar peduncle; these analyzes found lower MD (*p* < 0.2) in the anterior corona radiata, posterior corona radiata, tapetum, external capsule, superior and interior fronto‐occipital fasciculus, and pontine crossing tracts. Though these differences were not significant, they may also implicate aberrant white matter structural integrity in neonates with seizures and support the need for further research with larger sample sizes. The tapetum is part of the splenium of the corpus callosum forming the upper part of the lateral ventricle, while the external capsule and fronto‐occipital fasciculi are corticocortical association fibers implicated in language processing. The pontine crossing tracts and middle cerebellar peduncle contain afferent fibers to the cerebellum, implicated in coordination. Abnormal white matter structure in these regions may have important implications for neurodevelopmental outcomes in motor and language domains and may reflect broader network dysfunction.

We found no significant differences in the corpus callosum. This is in contrast to a prior study that showed a significantly lower MD in the corpus callosum of neonates with genetic epilepsy [30]. The prior finding of a significant difference in MD of the corpus callosum in neonates with genetic epilepsy versus healthy controls using ADC maps [30] may not have been redemonstrated in this study using FA and MD from DTI due to the differences in imaging analysis techniques; the corpus callosum may already be sufficiently myelinated as to obscure a difference between the groups, while the smaller association fibers undergoing more active myelination and pruning are more sensitive at this stage. Further, the entire corpus callosum was assessed on the left and right, rather than in specific callosal regions which may be more restricted to the regions affected by seizures. By assessing the entirety of the corpus callosum together, differences may have been obscured.

Further investigation is required to determine the nature of these changes, and whether they are related to seizure activity or to the underlying genetic diagnoses. Most of the neonates in this sample had epilepsy due to pathogenic variants in *KCNQ2*, which is the most common cause of neonatal‐onset genetic epilepsy [50]. It is unknown whether white matter is independently affected by genetic changes in channelopathies like *KCNQ2*‐related epilepsies; however, there is no current data to suggest this. There was no association between FA or MD and seizure burden assessed as number of seizures, seconds of seizure, or seizure burden category; however, this may be related to the small sample size rather than a true reflection of the impact of seizure burden on white matter. Future research should include larger sample sizes to better detect true seizure‐related white matter changes.

Regarding developmental outcomes, there was a significant difference in the mean FA of the right hippocampal portion of the cingulum in patients with genetic epilepsy with developmental delay vs. typical development, but no other regions met significance. The cingulum is an extensive white matter tract connecting the frontal, parietal, and temporal lobes, with the hippocampal portion of the cingulum implicated in memory. Abnormalities in the hippocampal portion of the cingulum have been described, with lower FA in adults with schizophrenia and anhedonia, and lower FA in Obsessive Compulsive Disorder [51]. The clinical significance of this finding in neonates is unclear and can be further explored in a larger sample.

Limitations of this study include the small cohort size, as well as the underlying genetic changes which may impact white matter development independently of seizure burden. Further study in a larger cohort of a different population of neonates is required to determine whether the white matter differences in neonates with seizures versus healthy controls are related to the underlying genetics or the seizures themselves. Further, FA and MD are not direct measures of myelination. Higher FA and lower MD may reflect aberrantly increased myelination, accelerated maturation, lack of normal axonal pruning, and/or other axonal abnormalities in the measured tracts [15, 16]. Thus, the specific nature of the white matter changes in this cohort is not known. Additionally, the clinical controls were obtained from a sample of NICU patients, and though there were no conditions known to affect white matter and no relevant clinical diagnoses, it is possible these neonates do not reflect a true healthy control population. There were, however, no significant differences between the clinical and research control neonates. Furthermore, future avenues of study would benefit from more well defined and validated measures of neurodevelopmental outcomes.

White matter microstructure is abnormal, with higher FA and lower MD, in neonates with seizures due to diverse genetic causes compared to healthy controls. This finding has high potential clinical relevance as aberrant white matter microstructure in this population may be an early biomarker for abnormal network formation and function. Further study is required to determine whether this can be a predictive measure applied clinically for neurocognitive and neurodevelopmental outcomes.

## Author Contributions

**Amanda G. Sandoval Karamian:** conceptualization, methodology, formal analysis, investigation, writing – original draft. **Tianjia Zhu:** software, formal analysis, investigation, writing – review and editing. **Hao Huang:** software, resources, writing – review and editing. **Shavonne L. Massey:** writing – review and editing, supervision. **Mark P. Fitzgerald:** writing – review and editing, supervision. **Darshana Parikh:** data curation. **Arastoo Vossough:** writing – review and editing, conceptualization, resources. **Ingo Helbig:** writing – review and editing, supervision. **Nicholas S. Abend:** writing – review and editing, supervision, conceptualization, methodology.

## Funding

This work was supported by the Pediatric Epilepsy Research Foundation and National Institutes of Health (Grant R01MH092535).

## Ethics Statement

This study was conducted with the approval of the Institutional Review Board of the Children's Hospital of Philadelphia, with an approved waiver of consent.

## Conflicts of Interest

The authors declare no conflicts of interest.

## Supporting information


**Table S1:** Mean fractional anisotropy (FA) values for cases and controls for 53 white matter tracts analyzed.
**Table S2:** Mean diffusivity (MD) values for cases and controls for 53 white matter tracts analyzed.
**Table S3:** Subgroup analysis of KCNQ2/KCNQ3 (*n* = 11) versus other genetic etiologies (*n* = 8).

## Data Availability

The data that support the findings of this study are available from the corresponding author upon reasonable request.
