# Supplementary material for: White Matter Microstructural Abnormalities in Neonatal Onset Genetic Epilepsy
Source: Ann Clin Transl Neurol. 2026 May 22:10.1002/acn3.70440. Online ahead of print. doi: 10.1002/acn3.70440 (PMC13394097; doi:10.1002/acn3.70440)
Supplement: Supplementary file 1 — Table S1: Mean fractional anisotropy (FA) values for cases and controls for 53 white matter tracts analyzed. Table S2: Mean diffusivity (MD) values for cases and controls for 53 white matter tracts analyzed. Table S3: Subgroup analysis of KCNQ2/KCNQ3 (n = 11) versus other genetic etiologies (n = 8). [file ACN3-9999-0-s001.docx]

**Supplemental Table 1.** Mean fractional anisotropy (FA) values for cases and controls for 53 white matter tracts analyzed.

| **Structure** | **Laterality** | **Mean (SD) FA Cases** | **Mean (SD) FA Controls** | **p-value** |
| --- | --- | --- | --- | --- |
| corpus callosum | left | 0.411 (0.044) | 0.407 (0.041) | 0.5891 |
| corpus callosum | right | 0.406 (0.044) | 0.405 (0.036) | 0.6096 |
| anterior limb of internal capsule | left | 0.294 (0.028) | 0.300 (0.029) | 0.5478 |
| anterior limb of internal capsule | right | 0.312 (0.025) | 0.316 (0.029) | 0.6941 |
| posterior limb of internal capsule | left | 0.497 (0.030) | 0.499 (0.029) | 0.9841 |
| posterior limb of internal capsule | right | 0.497 (0.029) | 0.503 (0.029) | 0.9543 |
| retrolenticular part of internal capsule | left | 0.414 (0.030) | 0.411 (0.030) | 0.8481 |
| retrolenticular part of internal capsule | right | 0.414 (0.028) | 0.409 (0.028) | 0.724 |
| anterior corona radiata | left | 0.229 (0.030) | 0.224 (0.025) | 0.2655 |
| anterior corona radiata | right | 0.215 (0.030) | 0.210 (0.027) | 0.2547 |
| superior corona radiata | left | 0.275 (0.035) | 0.258 (0.030) | **0.0166** |
| superior corona radiata | right | 0.266 (0.029) | 0.249 (0.026) | **0.0169** |
| posterior corona radiata | left | 0.288 (0.030) | 0.281 (0.030) | 0.2138 |
| posterior corona radiata | right | 0.294 (0.026) | 0.284 (0.031) | 0.1594 |
| cingulum cingulate gyrus | left | 0.280 (0.027) | 0.277 (0.035) | 0.5718 |
| cingulum cingulate gyrus | right | 0.259 (0.030) | 0.256 (0.031) | 0.3891 |
| cingulum hippocampal | left | 0.311 (0.029) | 0.310 (0.038) | 0.9849 |
| cingulum hippocampal | right | 0.312 (0.031) | 0.313 (0.037) | 0.6226 |
| fornix | left | 0.308 (0.027) | 0.306 (0.030) | 0.7149 |
| fornix | right | 0.315 (0.027) | 0.313 (0.027) | 0.6688 |
| stria terminalis | left | 0.377 (0.028) | 0.383 (0.033) | 0.7336 |
| stria terminalis | right | 0.361 (0.028) | 0.369 (0.028) | 0.7807 |
| tapetum | left | 0.287 (0.038) | 0.270 (0.043) | 0.0647 |
| tapetum | right | 0.288 (0.030) | 0.271 (0.039) | 0.0724 |
| superior longitudinal fasciculus | left | 0.228 (0.026) | 0.225 (0.025) | 0.3527 |
| superior longitudinal fasciculus | right | 0.221 (0.028) | 0.219 (0.027) | 0.409 |
| external capsule | left | 0.280 (0.022) | 0.271 (0.021) | 0.0772 |
| external capsule | right | 0.274 (0.022) | 0.266 (0.020) | 0.1145 |
| posterior thalamic radiata | left | 0.341 (0.033) | 0.343 (0.034) | 0.8245 |
| posterior thalamic radiata | right | 0.339 (0.032) | 0.334 (0.029) | 0.3527 |
| sagittal stratum | left | 0.281 (0.033) | 0.279 (0.030) | 0.428 |
| sagittal stratum | right | 0.278 (0.037) | 0.273 (0.030) | 0.2821 |
| cerebral peduncle | left | 0.398 (0.036) | 0.408 (0.038) | 0.4324 |
| cerebral peduncle | right | 0.394 (0.036) | 0.403 (0.033) | 0.6689 |
| superior fronto-occipital fasciculus | left | 0.257 (0.036) | 0.254 (0.035) | 0.9058 |
| superior fronto-occipital fasciculus | right | 0.244 (0.033) | 0.237 (0.036) | 0.3876 |
| inferior fronto-occipital fasciculus | left | 0.297 (0.027) | 0.292 (0.027) | 0.2624 |
| inferior fronto-occipital fasciculus | right | 0.293 (0.030) | 0.287 (0.026) | 0.3957 |
| corticospinal tract | left | 0.288 (0.040) | 0.294 (0.034) | 0.2376 |
| corticospinal tract | right | 0.282 (0.041) | 0.286 (0.031) | 0.6400 |
| superior cerebellar peduncle | left | 0.315 (0.023) | 0.321 (0.028) | 0.6960 |
| superior cerebellar peduncle | right | 0.321 (0.024) | 0.325 (0.031) | 0.9976 |
| middle cerebellar peduncle | left | 0.320 (0.037) | 0.318 (0.038) | 0.1478 |
| middle cerebellar peduncle | right | 0.319 (0.043) | 0.322 (0.035) | 0.9872 |
| inferior cerebellar peduncle | left | 0.288 (0.038) | 0.291 (0.036) | 0.8442 |
| inferior cerebellar peduncle | right | 0.290 (0.043) | 0.297 (0.039) | 0.9039 |
| pontine crossing tract | left | 0.259 (0.028) | 0.263 (0.031) | 0.4748 |
| pontine crossing tract | right | 0.250 (0.035) | 0.245 (0.029) | 0.8381 |
| uncinate fasciculus | left | 0.253 (0.041) | 0.248 (0.029) | 0.4324 |
| uncinate fasciculus | right | 0.229 (0.046) | 0.228 (0.038) | 0.3689 |
| medial lemniscus | left | 0.288 (0.034) | 0.293 (0.029) | 0.6013 |
| medial lemniscus | right | 0.290 (0.039) | 0.289 (0.028) | 0.8058 |
| anterior commissure | left | 0.264 (0.030) | 0.266 (0.035) | 0.8428 |
| anterior commissure | right | 0.260 (0.028) | 0.257 (0.034) | 0.7495 |

Significant p-values <0.05 are in bold.

**Supplemental Table 2.** Mean diffusivity (MD) values for cases and controls for 53 white matter tracts analyzed.

| Structure | Laterality | Mean (SD) MD Cases | Mean (SD) MD Controls | p-value |
| --- | --- | --- | --- | --- |
| corpus callosum | left | 0.00100 (0.00013) | 0.00101 (0.00012) | 0.3443 |
| corpus callosum | right | 0.00101 (0.00013) | 0.00101 (0.00012) | 0.3095 |
| anterior limb of internal capsule | left | 0.00094 (0.00008) | 0.00094 (0.00010) | 0.8368 |
| anterior limb of internal capsule | right | 0.00093 (0.00008) | 0.00093 (0.00010) | 0.7443 |
| posterior limb of internal capsule | left | 0.00071 (0.00007) | 0.00071 (0.00008) | 0.9914 |
| posterior limb of internal capsule | right | 0.00071 (0.00006) | 0.00071 (0.00008) | 0.9670 |
| retrolenticular part of internal capsule | left | 0.00086 (0.00007) | 0.00086 (0.00009) | 0.7649 |
| retrolenticular part of internal capsule | right | 0.00086 (0.00007) | 0.00086 (0.00009) | 0.6141 |
| anterior corona radiata | left | 0.00120 (0.00012) | 0.00123 (0.00013) | 0.1243 |
| anterior corona radiata | right | 0.00122 (0.00012) | 0.00126 (0.00012) | 0.1058 |
| superior corona radiata | left | 0.00109 (0.00014) | 0.00113 (0.00012) | 0.0957 |
| superior corona radiata | right | 0.00109 (0.00012) | 0.00114 (0.00012) | **0.0494** |
| posterior corona radiata | left | 0.00108 (0.00012) | 0.00109 (0.00011) | 0.3041 |
| posterior corona radiata | right | 0.00108 (0.00011) | 0.00110 (0.00012) | 0.1472 |
| cingulum cingulate gyrus | left | 0.00103 (0.00008) | 0.00105 (0.00009) | 0.2416 |
| cingulum cingulate gyrus | right | 0.00106 (0.00008) | 0.00107 (0.00009) | 0.2162 |
| cingulum hippocampal | left | 0.00097 (0.00007) | 0.00100 (0.00012) | 0.2196 |
| cingulum hippocampal | right | 0.00096 (0.00009) | 0.00098 (0.00012) | 0.3060 |
| fornix | left | 0.00105 (0.00010) | 0.00105 (0.00010) | 0.5132 |
| fornix | right | 0.00107 (0.00011) | 0.00106 (0.00012) | 0.4274 |
| stria terminalis | left | 0.00091 (0.00008) | 0.00090 (0.00008) | 0.7288 |
| stria terminalis | right | 0.00094 (0.00009) | 0.00095 (0.00009) | 0.2898 |
| tapetum | left | 0.00126 (0.00015) | 0.00130 (0.00016) | 0.1152 |
| tapetum | right | 0.00123 (0.00012) | 0.00127 (0.00015) | 0.0998 |
| superior longitudinal fasciculus | left | 0.00124 (0.00012) | 0.00125 (0.00011) | 0.5055 |
| superior longitudinal fasciculus | right | 0.00128 (0.00013) | 0.00129 (0.00012) | 0.3729 |
| external capsule | left | 0.00100 (0.00008) | 0.00103 (0.00010) | 0.1525 |
| external capsule | right | 0.00101 (0.00007) | 0.00104 (0.00010) | 0.1557 |
| posterior thalamic radiata | left | 0.00108 (0.00010) | 0.00109 (0.00012) | 0.5948 |
| posterior thalamic radiata | right | 0.00107 (0.00010) | 0.00107 (0.00010) | 0.2707 |
| sagittal stratum | left | 0.00115 (0.00012) | 0.00115 (0.00013) | 0.4208 |
| sagittal stratum | right | 0.00114 (0.00012) | 0.00115 (0.00013) | 0.4379 |
| cerebral peduncle | left | 0.00085 (0.00013) | 0.00085 (0.00011) | 0.8888 |
| cerebral peduncle | right | 0.00086 (0.00014) | 0.00087 (0.00012) | 0.5352 |
| superior fronto-occipital fasciculus | left | 0.00109 (0.00013) | 0.00109 (0.00012) | 0.4853 |
| superior fronto-occipital fasciculus | right | 0.00111 (0.00013) | 0.00113 (0.00013) | 0.1839 |
| inferior fronto-occipital fasciculus | left | 0.00100 (0.00010) | 0.00102 (0.00011) | 0.3800 |
| inferior fronto-occipital fasciculus | right | 0.00100 (0.00011) | 0.00102 (0.00011) | 0.1911 |
| corticospinal tract | left | 0.00102 (0.00024) | 0.00103 (0.00025) | 0.4807 |
| corticospinal tract | right | 0.00110 (0.00025) | 0.00107 (0.00025) | 0.8132 |
| superior cerebellar peduncle | left | 0.00081 (0.00007) | 0.00081 (0.00009) | 0.7784 |
| superior cerebellar peduncle | right | 0.00080 (0.00008) | 0.00079 (0.00010) | 0.8991 |
| middle cerebellar peduncle | left | 0.00096 (0.00014) | 0.00095 (0.00014) | 0.8528 |
| middle cerebellar peduncle | right | 0.00097 (0.00013) | 0.00094 (0.00013) | 0.7424 |
| inferior cerebellar peduncle | left | 0.00093 (0.00016) | 0.00090 (0.00015) | 0.6807 |
| inferior cerebellar peduncle | right | 0.00089 (0.00014) | 0.00088 (0.00013) | 0.7201 |
| pontine crossing tract | left | 0.00090 (0.00017) | 0.00095 (0.00017) | 0.1403 |
| pontine crossing tract | right | 0.00094 (0.00021) | 0.00100 (0.00019) | 0.1023 |
| uncinate fasciculus | left | 0.00106 (0.00011) | 0.00106 (0.00012) | 0.6451 |
| uncinate fasciculus | right | 0.00108 (0.00012) | 0.00110 (0.00013) | 0.3637 |
| medial lemniscus | left | 0.00083 (0.00016) | 0.00084 (0.00014) | 0.5020 |
| medial lemniscus | right | 0.00085 (0.00016) | 0.00086 (0.00015) | 0.6214 |
| anterior commissure | left | 0.00089 (0.00008) | 0.00090 (0.00010) | 0.6832 |
| anterior commissure | right | 0.00091 (0.00008) | 0.00092 (0.00010) | 0.4793 |

Significant p-values <0.05 are in bold.

**Supplemental Table 3.** Subgroup analysis of KCNQ2/KCNQ3 (n=11) versus other genetic etiologies (n=8).

| **Structure** | **Laterality** | **Mean (SD) FA KCNQ2/KCNQ3** | **Mean (SD) FA Other genetic etiologies** | **p-value** |
| --- | --- | --- | --- | --- |
| corpus callosum | left | 0.409 (0.027) | 0.415 (0.063) | 0.945 |
| corpus callosum | right | 0.400 (0.035) | 0.414 (0.056) | 0.932 |
| posterior limb of internal capsule | left | 0.503 (0.025) | 0.489 (0.035) | 0.575 |
| posterior limb of internal capsule | right | 0.496 (0.027) | 0.498 (0.034) | 0.932 |
| superior corona radiata | left | 0.271 (0.032) | 0.281 (0.040) | 0.212 |
| superior corona radiata | right | 0.265 (0.025) | 0.268 (0.035) | 0.575 |
| posterior corona radiata | left | 0.288 (0.029) | 0.289 (0.033) | 0.254 |
| posterior corona radiata | right | 0.297 (0.028) | 0.289 (0.025) | 0.575 |
| tapetum | left | 0.280 (0.033) | 0.297 (0.044) | 0.575 |
| tapetum | right | 0.284 (0.025) | 0.294 (0.036) | 0.575 |
| external capsule | left | 0.281 (0.016) | 0.279 (0.031) | 0.575 |
| external capsule | right | 0.273 (0.020) | 0.276 (0.027) | 0.675 |
| superior cerebellar peduncle | left | 0.312 (0.019) | 0.315 (0.033) | 0.932 |
| superior cerebellar peduncle | right | 0.320 (0.023) | 0.324 (0.035) | 0.945 |
| middle cerebellar peduncle | left | 0.317 (0.034) | 0.318 (0.044) | 0.212 |
| middle cerebellar peduncle | right | 0.319 (0.040) | 0.323 (0.038) | 0.575 |
| inferior cerebellar peduncle | left | 0.290 (0.036) | 0.291 (0.036) | 0.575 |
| inferior cerebellar peduncle | right | 0.291 (0.039) | 0.294 (0.039) | 0.731 |
| pontine crossing tract | left | 0.252 (0.030) | 0.260 (0.038) | 0.804 |
| pontine crossing tract | right | 0.249 (0.031) | 0.248 (0.035) | 0.932 |
